# Facile control of nanoporosity in Cellulose Acetate using Nickel(II) nitrate additive and water pressure treatment for highly efficient battery gel separators

**DOI:** 10.1038/s41598-017-01399-8

**Published:** 2017-04-28

**Authors:** Woong Gi Lee, Do Hyeong Kim, Woo Cheol Jeon, Sang Kyu Kwak, Seok Ju Kang, Sang Wook Kang

**Affiliations:** 1 Department of Chemistry, Sangmyung University, Seoul, 110-743 Republic of Korea; 2 School of Energy and Chemical Engineering, Ulsan National Institute of Science and Technology (UNIST), Ulsan, 689-798 Republic of Korea

## Abstract

We succeed in fabricating nearly straight nanopores in cellulose acetate (CA) polymers for use as battery gel separators by utilizing an inorganic hexahydrate (Ni(NO_3_)_2_·6H_2_O) complex and isostatic water pressure treatment. The continuous nanopores are generated when the polymer film is exposed to isostatic water pressure after complexing the nickel(II) nitrate hexahydrate (Ni(NO_3_)_2_·6H_2_O) with the CA. These results can be attributed to the manner in which the polymer chains are weakened because of the plasticization effect of the Ni(NO_3_)_2_·6H_2_O that is incorporated into the CA. Furthermore, we performed extensive molecular dynamics simulation for confirming the interaction between electrolyte and CA separator. The well controlled CA membrane after water pressure treatment enables fabrication of highly reliable cell by utilizing 2032-type coin cell structure. The resulting cell performance exhibits not only the effect of the physical morphology of CA separator, but also the chemical interaction of electrolyte with CA polymer which facilitates the Li-ion in the cell.

## Introduction

Porous materials – and in particular nanoporous membranes – have been widely used in various nano- and bio-technology applications such as gas storage, filters, battery separators, polymer electrolyte membranes, polymer support, and water treatment or purification^1–8^. Specifically for battery separators, porous organic materials have become promising candidates for highly efficient lithium (Li)-ion or lithium-metal battery applications because of the fact that their morphologies can be easily tuned by using smart functional materials and chemistry^9–12^. Thus, it has been intensively studied to further improve battery performance with advanced anode and cathodes materials^13–15^. For example, previous work by T. C. Mike Chung research group suggested a new class of polyethylene-based anion exchange membrane (PE-AEMs) as separator of lithium ion battery. The PE-AEMs has contained flexible ammonium chloride (−NR_3_^+^Cl^−^) and a cross-linking PE network structure. The properties of this membrane show high thermal stability, including adequate water swelling, and exceptionally high ionic-conductivity of 119.6 mS/cm in 2N HCl solution and 78.8 mS/cm in 2N HCl-0.2N CuCl solution^16^. Also, Zhuyi Wang research group suggested an organic-inorganic hybrid multilayer battery separator using by layer-by-layer (LbL) self-assembly process. The formation of functional ultrathin multilayer on PE separator exhibited high performance battery properties^17, 18^. In addition, the porous separators of battery have been fabricated through a variety of methods, such as thermally induced phase separation, phase inversion, and track-etching, anodization and blade casting process^19–29^. However, certain vexing problems accompany these methods, which include complicated procedures and high-cost processes for mass production; such obstacles are not desirable in battery separator applications.

In order to remedy these issues, simple, low-cost, energy-efficient, and environmentally friendly methods for generating nanopores are needed to generate the polymer separators. Recently, cellulose and its derivative polymer matrix have been studied on account of their various advantages such as high strength, durability, high thermal stability, good biocompatibility, relatively low cost, low density, and good mechanical stability^30^. However, although such nanopores possess these significant intrinsic advantages, it is still difficult to utilize the nanopores and to obtain a large fabrication area.

In this study, we develop a simple, low-cost, energy-efficient, and environmentally friendly method for fabricating nearly straight nanopores in a thermally stable cellulose acetate (CA) polymer matrix by combining an inorganic complex with isostatic water pressure treatment. The straight-type CA separator and gelation phenomena allow us to achieve stable Li-ion transport. The molecular dynamic (MD) simulation also reveals that electrolytes can easily diffuse uptake into the CA separator, resulting in decrease of the resistance in the battery cell. Furthermore, we demonstrate a Li-ion half-cell battery with a LTO electrode. The half cell with a well-controlled CA separator exhibits stable electrochemistry without any serious degradation in battery performance.

## Results and Discussion

### Surface morphology

**Figure 1:**
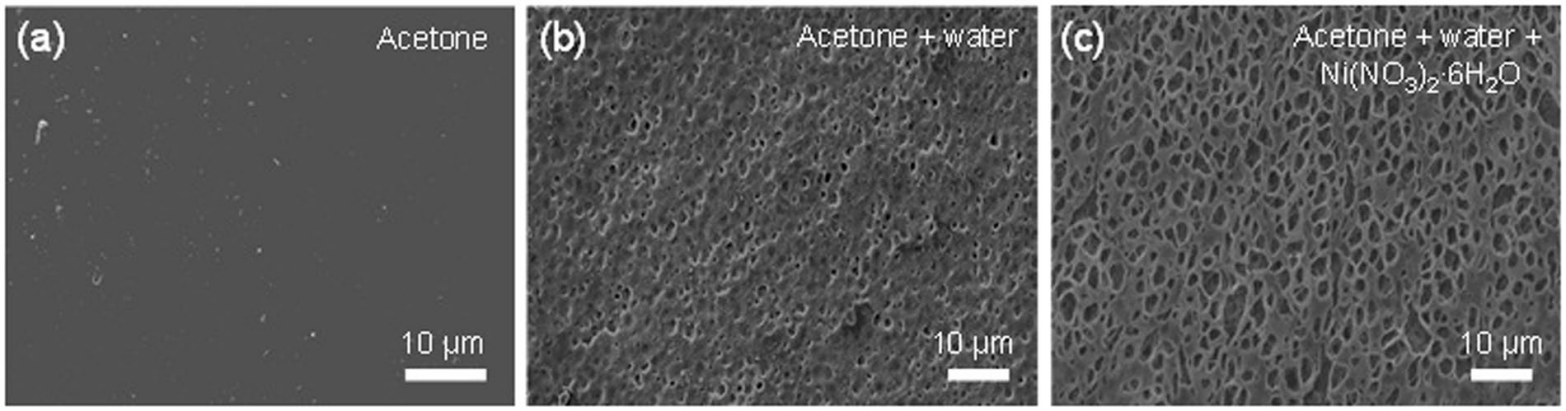
Scanning electron microscopy (SEM) images. (**a**) Pores observed in a neat cellulose acetate (CA) polymer matrix in acetone. (**b**) Neat CA polymer matrix in acetone/water (w/w 8:2). (**c**) 1:0.23 CA/Ni(NO_3_)_2_·6H_2_O polymer matrix in acetone/water (w/w 8:2).

Scanning electron microscopy (SEM) was used to investigate the pores generated in the CA polymer matrix using pure acetone, acetone/water (w/w 8:2), and acetone/water (w/w 8:2) with a Ni(NO_3_)_2_·6H_2_O additive. Figure 1(a) shows the surface morphology of neat CA, which was dissolved in pure acetone. The SEM image clearly demonstrates that there are no pores on the surface of the CA polymer dissolved in pure acetone. By way of contrast, for the CA polymer matrix dissolved in the acetone/water co-solvent, pores were generated on the surface, as shown in Fig. 1(b). The pores had a radius of approximately 1 μm and were homogenously distributed on the surface mainly owing to the presence of the high boiling point of water molecules in the solution during the film formation.

Although the acetone/water co-solvent generates pores on the CA polymer matrix, it is still difficult to control the size and porosity of the pores during membrane formation. Therefore, in order to control the pore size of the CA membrane, we introduced a new approach that incorporates Ni(NO_3_)_2_·6H_2_O into the acetone/water blend solvent. The resulting SEM image shows that the pore size and porosity of the polymer matrix dramatically increased on the CA polymer surface. It should be noted that there are two major plausible explanations for why the larger pores were formed in the CA membrane. i) The solvated Ni(NO_3_)_2_·6H_2_O aggregates in the polymer matrix during solidification, while the volatile acetone rapidly evaporates; this results in the remaining Ni(NO_3_)_2_·6H_2_O aggregates forming well-defined pores in the CA polymer matrix. ii) There is strong molecular-level ionic association between Ni, nitrate, and water molecules. This strong interaction retards the evaporation of water molecules in the CA polymer matrix, which results in the formation of pores on the surface.

### Pressure effect on polymer matrix

**Figure 2:**
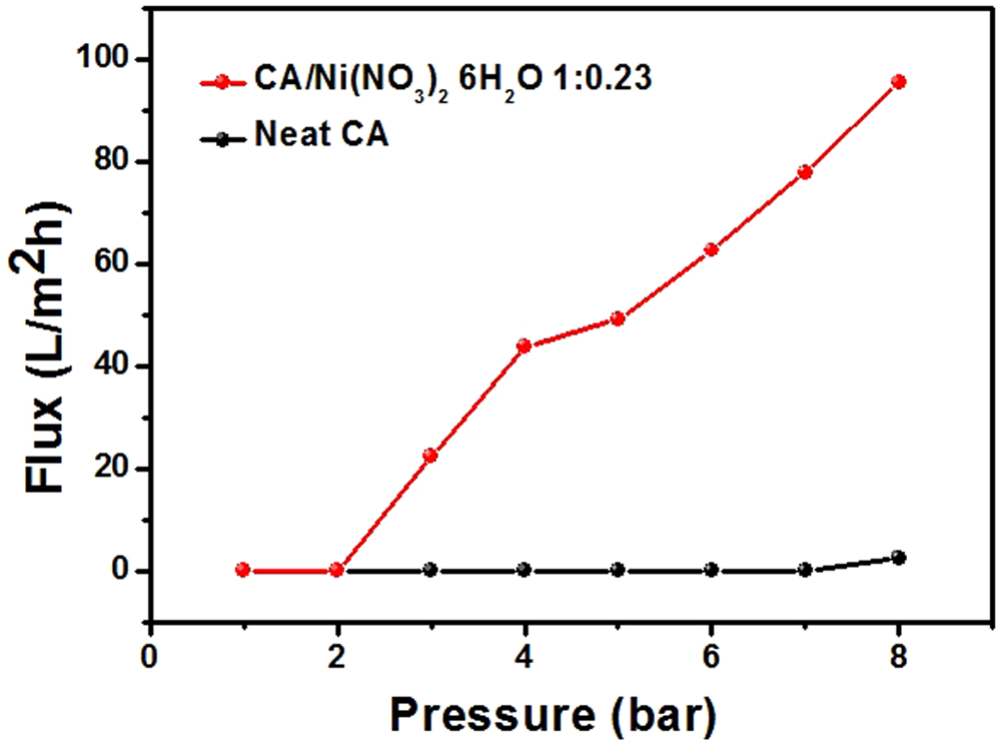
Flux measured through neat cellulose acetate (CA) and CA with Ni(NO_3_)_2_·6H_2_O at various water pressures.

**Figure 3:**
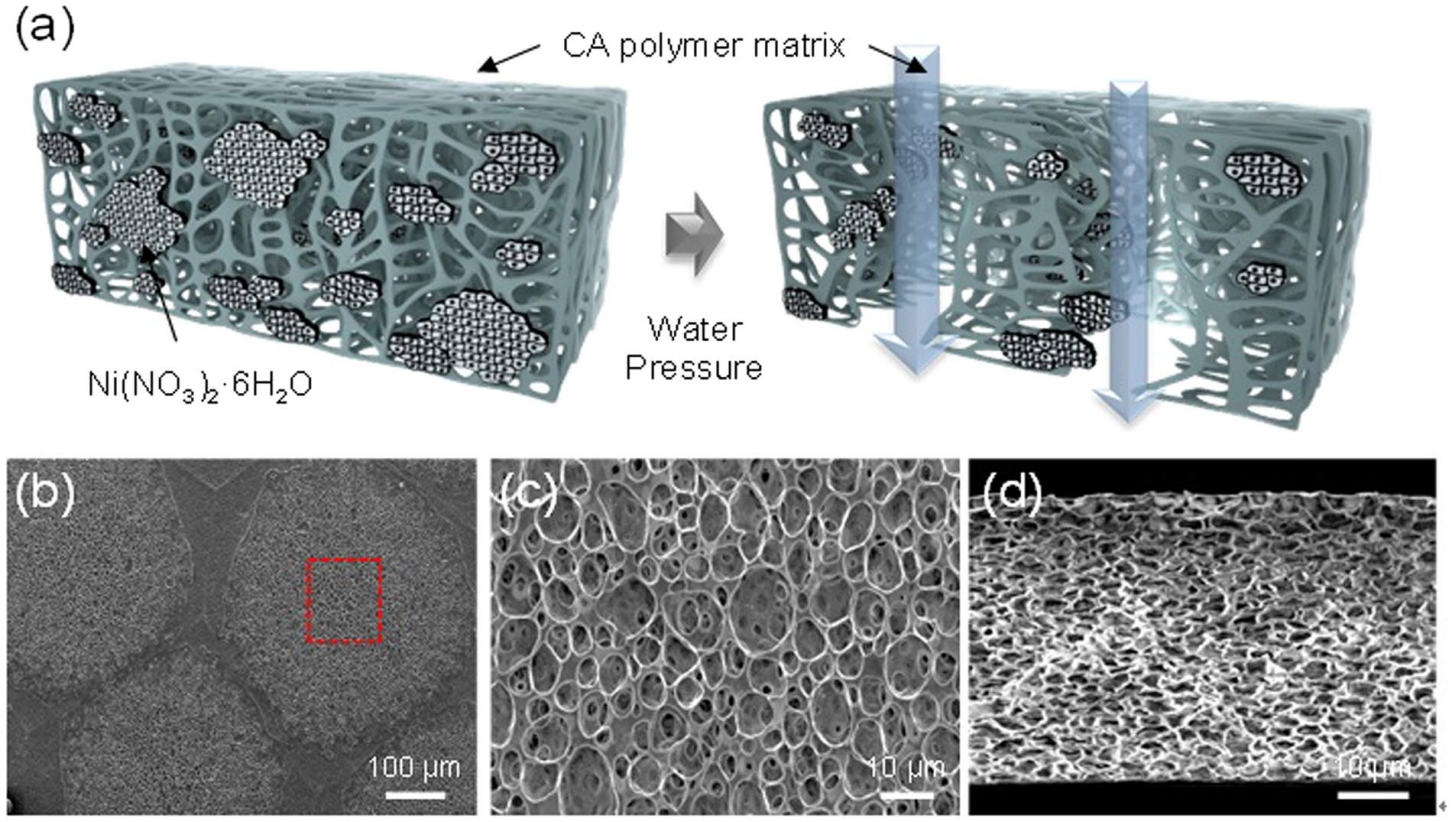
(**a**) Schematic diagram of the proposed nanoporous (CA) polymer. Top view (SEM) images of CA surface morphology observed in 1:0.23 CA/ Ni(NO_3_)_2_·6H_2_O in acetone/water (w/w 8:2) at water pressures of 5 bar (**b**). The red dashed squares in (**b**) indicate the magnified areas shown in panes, respectively (**c**). Cross-sectional views of the samples subjected to water pressures of 5 bar (**d**).

To further increase the pore size, 1:0.23 CA/ Ni(NO_3_)_2_·6H_2_O polymer matrix films were examined as a function of isostatic water pressure, which ranged from 2 to 8 bar. The dried polymer matrix containing Ni(NO_3_)_2_·6H_2_O was placed in water-treatment equipment and then subjected to water pressures ranging from 2 to 8 bar. Figure 2 shows a graph of the flux as a function of water pressure after the water pressure treatment. The graph illustrates that there is no significant water flux until the water pressure reaches 2 bar. From this point, for water pressures beyond 3 bar, the flux monotonically increases as a function of water pressure. The primary explanation for the increased pore size as a function of water pressure is that the CA chains, which were weakened by the Ni(NO_3_)_2_·6H_2_O solvate, were torn by the high pressure. This results in a well-defined channel, as shown in Fig. 3(a). It was thought that the mobility of Ni(NO_3_)_2_·6H_2_O in CA polymer could provide the space to be freely moved for polymer chains, resulting in the plasticization effect to increase the free volume. Thus, this plasticization effect plays a critical role in generating interconnected pores. Figure 3(b–d) shows SEM images of the pores generated in 1:0.23 CA/ Ni(NO_3_)_2_·6H_2_O matrices at water pressures of 5 bar and 8 bar (Supplementary Fig. S1), respectively.

### Plasticization effect

**Figure 4:**
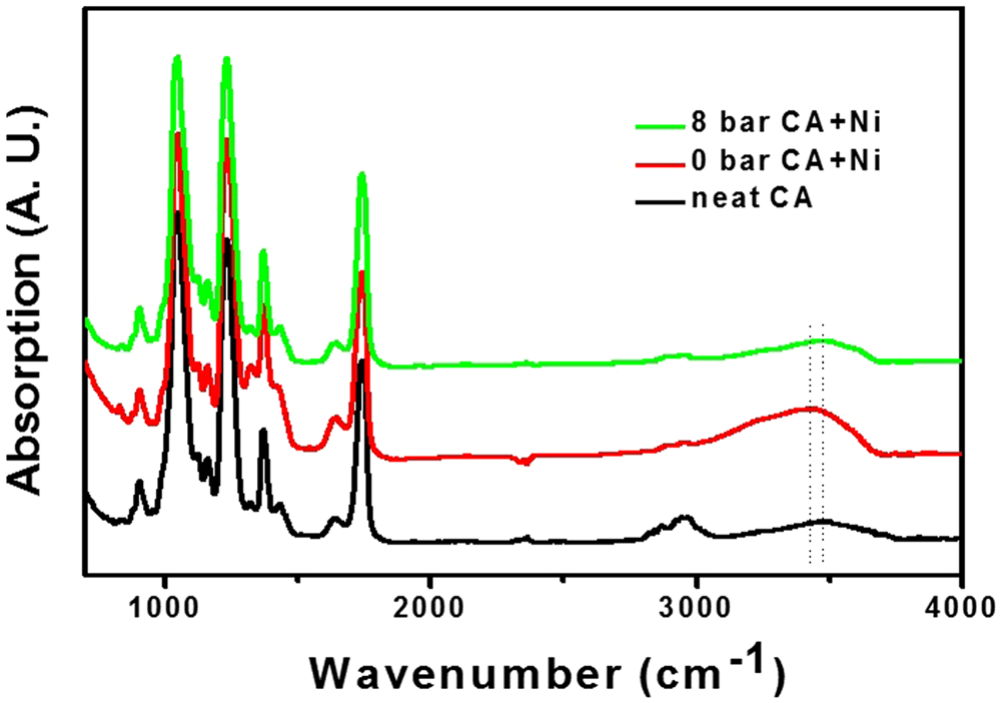
Fourier transform infrared (FT-IR) spectra of neat cellulose acetate (CA) and the 1:0.23 CA/ Ni(NO_3_)_2_·6H_2_O polymer matrix at water pressures of 0 bar and 8 bar.

In order to further understand the plasticization effect of Ni(NO_3_)_2_·6H_2_O solvates with CA chains during the water treatment process, we obtained FT-IR spectra by using a VERTEX 70 FT-IR spectrometer (Bruker Optics Inc.). Figure 4 shows the FT-IR spectra of neat CA as well as 1:0.23 CA/Ni(NO_3_)_2_·6H_2_O at water pressures of 0 bar and 8 bar. The neat CA polymer matrix sample exhibits the characteristic IR peak at 3478 cm^−1^, which corresponds to the hydroxyl group of the CA polymer. By way of contrast, the sample with Ni(NO_3_)_2_·6H_2_O solvates shows representative absorption bands for CA/Ni(NO_3_)_2_·6H_2_O at 3422 cm^−1^ because of the abundance of H_2_O molecules in Ni(NO_3_)_2_·6H_2_O, which cause the intensity of the OH adsorption peak to increase. The subsequent water pressure to the CA polymer sample at 8 bar. The peak observed at the 3422 cm^−1^ for CA/Ni(NO_3_)_2_·6H_2_O shifted to 3478 cm^−1^, indicating that a considerable amount of Ni(NO_3_)_2_·6H_2_O was removed by the high-water-pressure treatment.

### Porosity of the CA polymer

**Figure 5:**
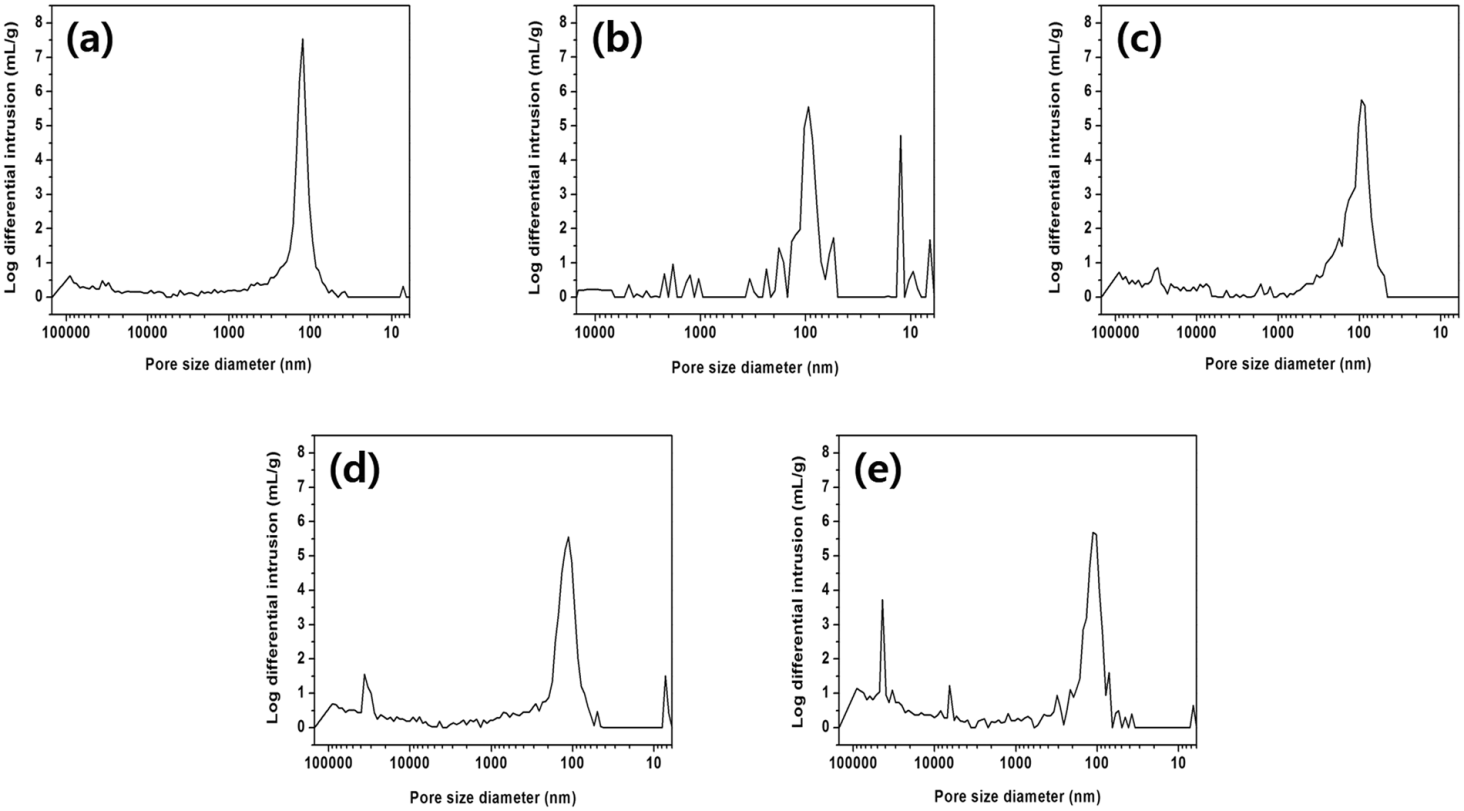
Porosimeter data. (**a**) Porosity in a neat cellulose acetate (CA) polymer matrix at a water pressure of 0 bar. Porosity in a 1:0.23 CA/Ni(NO_3_)_2_ polymer matrix at water pressures of (**b**) 0 bar, (**c**) 2 bar, (**d**) 5 bar, and (**e**) 8 bar.

In order to quantitatively measure the increased pore size and porosity of the CA polymer matrix, we utilized a mercury porosimeter (Autopore IV9500 model, Micromeritics Inc.). Figure 5(a) and (b) show the pore size of the CA polymer matrix without and with the Ni(NO_3_)_2_ additive. The dominant pore size of the CA polymer without Ni(NO_3_)_2_ shows a sharp peak at 120 nm; However, the sample with the Ni(NO_3_)_2_ additive exhibits a broad band around a few hundred nanometers, implying that the Ni(NO_3_)_2_ additive broadens the pore size in the CA polymer matrix. We observed larger pore sizes and volumes from water pressure samples with the Ni(NO_3_)_2_ additive, as shown in Fig. 5(c–e). We can conclude from these data that the pore size and volume increased considerably from the water pressure treatment for the polymer matrix that contained the Ni(NO_3_)_2_ additive.

### Interaction between CA polymer and electrolyte

To apply the CA polymer matrix in battery separator, the interacting preference between CA and electrolytes (ethylene carbonate (EC), and diethyl carbonate (DEC)) were further investigated by Flory-Huggins (FH) interaction parameter^31, 32^ (*χ*) calculated by using Hildebrand solubility parameter^33^ (δ) (Supplementary Note 1 in Supplementary information). Three different temperatures of 298 K, 323 K and 363 K were considered representing room temperature, battery heating temperature^34^, and simulation temperature. According to Flory-Huggins theory^31, 32^, when *χ* value goes to zero, the constituent materials have better compatibility and higher affinity. In other words, smaller *χ* value indicates that two constituent materials are thermodynamically more miscible and this phenomenon is decisively referred by similar *δ* values of the materials. Note that the last temperature was set to expedite molecular dynamics (MD) for a rapid observation of the time-dependent phase of the system. Table 1 shows calculated Hildebrand solubility parameters and FH parameters among CA, EC and DEC. χ_CA-EC_ was decreased as temperature was increased. It indicates that CA and EC are more miscible at high temperature. On the other hand, χ_CA-DEC_ followed an opposite behavior, where CA and DEC are more immiscible at elevated temperature. Finally, χ_EC-DEC_ showed the same behavior as χ_CA-EC_, yet the larger values compared to χ_CA-EC_ and χ_CA-DEC_ were indicative of partial unmixing.

**Table 1:** Hildebrand solubility parameters (*δ* s) and Flory-Huggins interaction parameters (*χ*_ij_ *’*s).

Temperature (K)	*δ*_CA_ (J/cm^3^)^0.5^	*δ*_EC_ (J/cm^3^)^0.5^	*δ*_DEC_ (J/cm^3^)^0.5^	*χ*_CA-EC_	χ_CA-DEC_	*χ*_EC-DEC_
**298**	26.2(24.5)	30.0(29.6)	18.7(18.0)	0.37	2.81	6.30
**323**	25.9	29.3	18.1	0.27	2.91	5.92
**363**	25.6	28.2	17.1	0.16	3.14	5.41

The values in parentheses are from Hansen^37^ and the average error percent is about ~4.2%.

**Figure 6:**
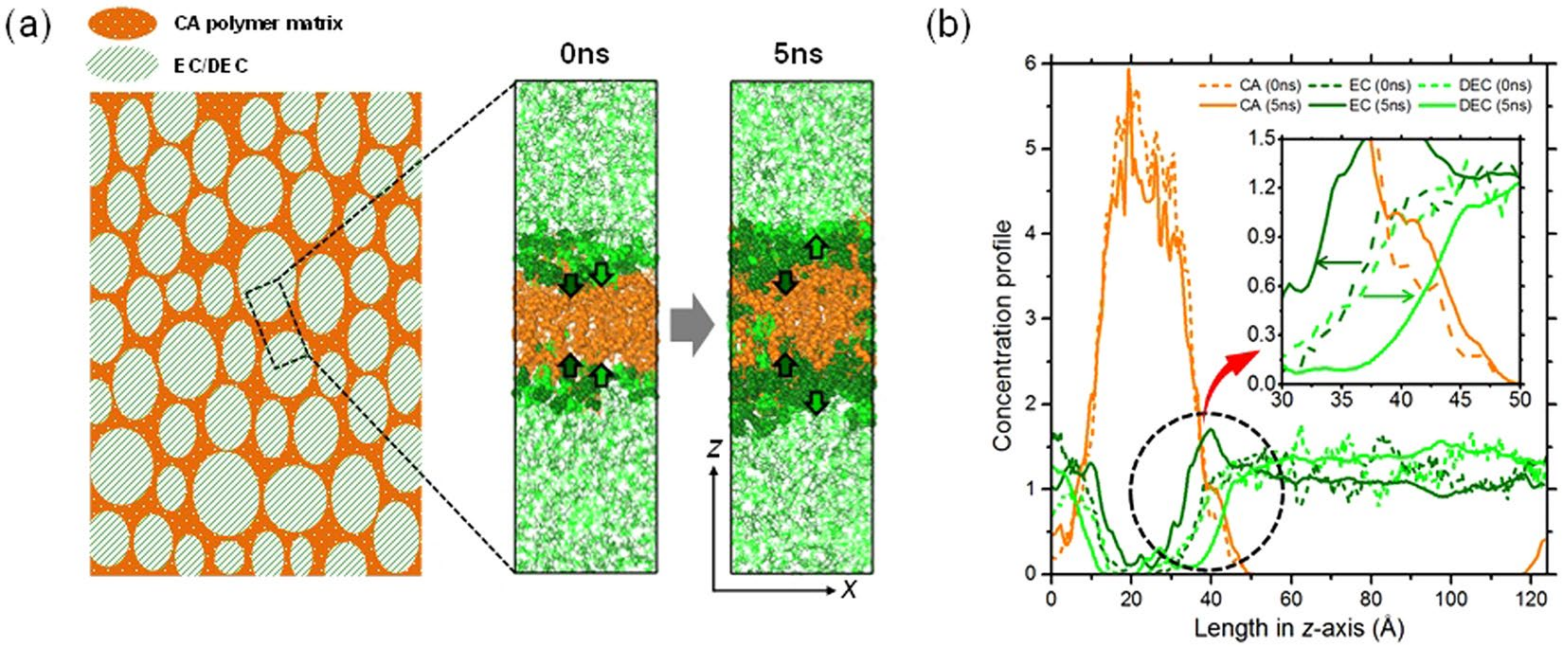
(**a**) Schematic diagrams of cross-section of CA polymer matrix with EC/DEC liquid mixture. The vol% ratio of CA to EC/DEC is 20:80, where that of EC to DEC is 50:50. Snapshots of MD simulations, which are presented on the left, show the initial (0 ns) and final (5 ns) states of the system. Dark green and light green arrows indicate the diffusion of EC and DEC molecules, respectively. (**b**) Concentration profiles of CA, EC, and DEC of the initial (0 ns) and final (5 ns) states of the layered system. Orange, dark green and light green colors represent CA, EC, and DEC molecules, respectively.

**Figure 7:**
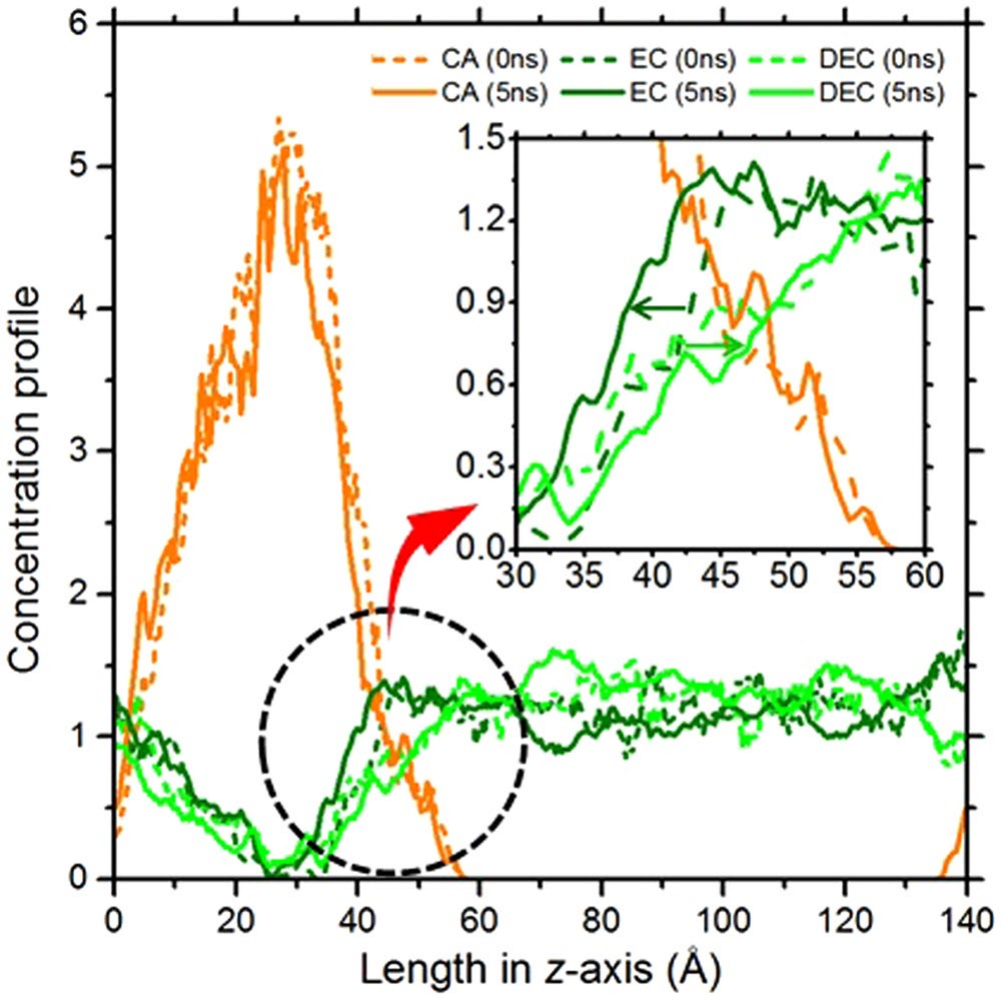
Concentration profiles of CA, EC, and DEC of the initial (0 ns) and final (5 ns) states of the layered system.

Based on the miscibility predicted by the FH parameters, the layered model system of CA and EC/DEC was constructed to investigate the intermixing dynamics of the components. Figure 6 shows the model system and the results of MD simulation at 363 K and 1 atm (see MD simulation in Methods). At early stage, both EC and DEC started diffusing into CA together. As time passed, EC diffused into CA while DEC was away from CA as predicted by the characteristic behavior of FH parameters. Note that the same phenomenon was observed with 30mer CA (Fig. 7). More notably, the diffusivity of CA, which was obtained from additional MD simulation (Supplementary Note 2 in Supplementary information), revealed that the diffusivity in the layered system (~1.21 × 10^−14^cm^2^/s) was two orders of magnitude greater than that in the bulk phase (~1.54 × 10^−16^ cm^2^/s). The result indicated that the presence of EC/DEC induced the free movement of CA. Thus, we conjecture that the gel-like phase of CA might appear due to the active inter-diffusion of EC/DEC during the battery operation.

### Thermal stability of CA polymer

**Figure 8:**
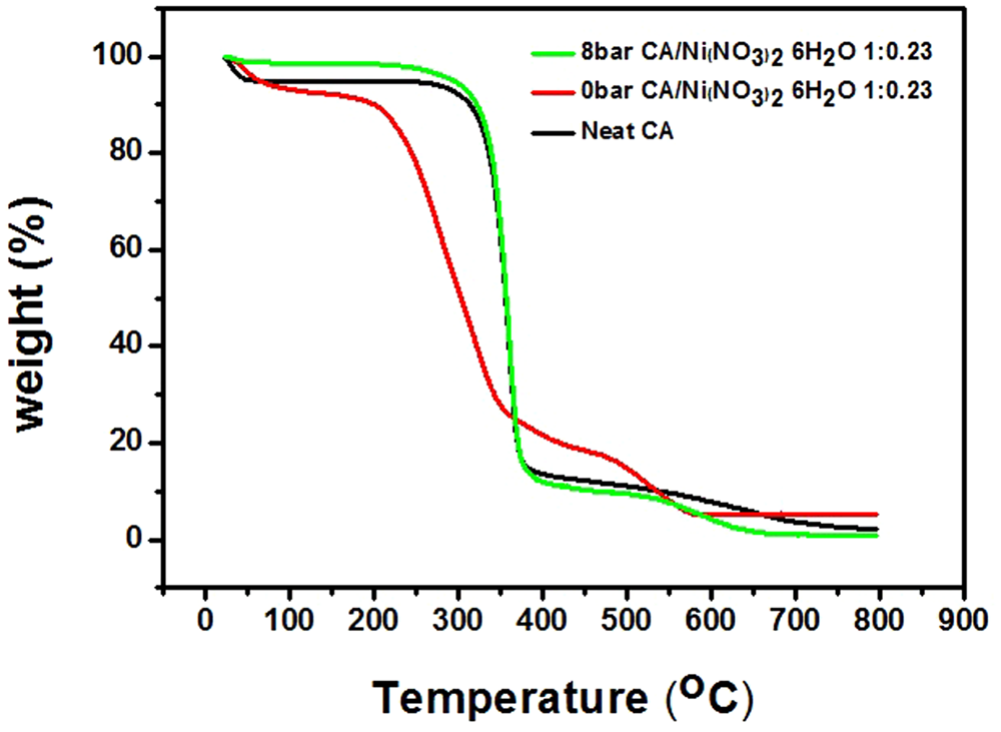
Thermogravimetric analysis (TGA) of neat cellulose acetate (CA) and CA/ Ni(NO_3_)_2_·6H_2_O polymer matrix at water pressures of 0 bar and 8 bar.

TGA was carried out to confirm the thermal stability of the porous polymer matrix using a Universal V4.5 A (TA Instruments). Figure 8 shows that about 90 wt% of neat CA and CA incorporated with Ni(NO_3_)_2_·6H_2_O at 8 bar were decomposed at around 300 °C. On the other hand, about 80 wt% of CA with Ni(NO_3_)_2_·6H_2_O at 0 bar was decomposed between 200 and 350 °C, and 2 wt% was decomposed between 350 and 550 °C. The boiling point of Ni(NO_3_)_2_·6H_2_O is 136.7 °C. It was estimated that polymer chains were loosened by the solvated Ni(NO_3_)_2_·6H_2_O in the polymer chains. Thus, the loss of about 80 wt% CA with Ni(NO_3_)_2_·6H_2_O can be attributed to the degradation of solvated Ni(NO_3_)_2_·6H_2_O and the loosened polymer chain. However, the thermal stability of the polymer matrix increased as a result of the water treatment. This increase in thermal stability can be explained by the fact that most Ni(NO_3_)_2_∙6H_2_O was removed from the CA polymer. Furthermore, it was confirmed that the little remaining Ni(NO_3_)_2_∙6H_2_O was decomposed above 400 °C.

### Electrochemical characteristic

**Figure 9:**
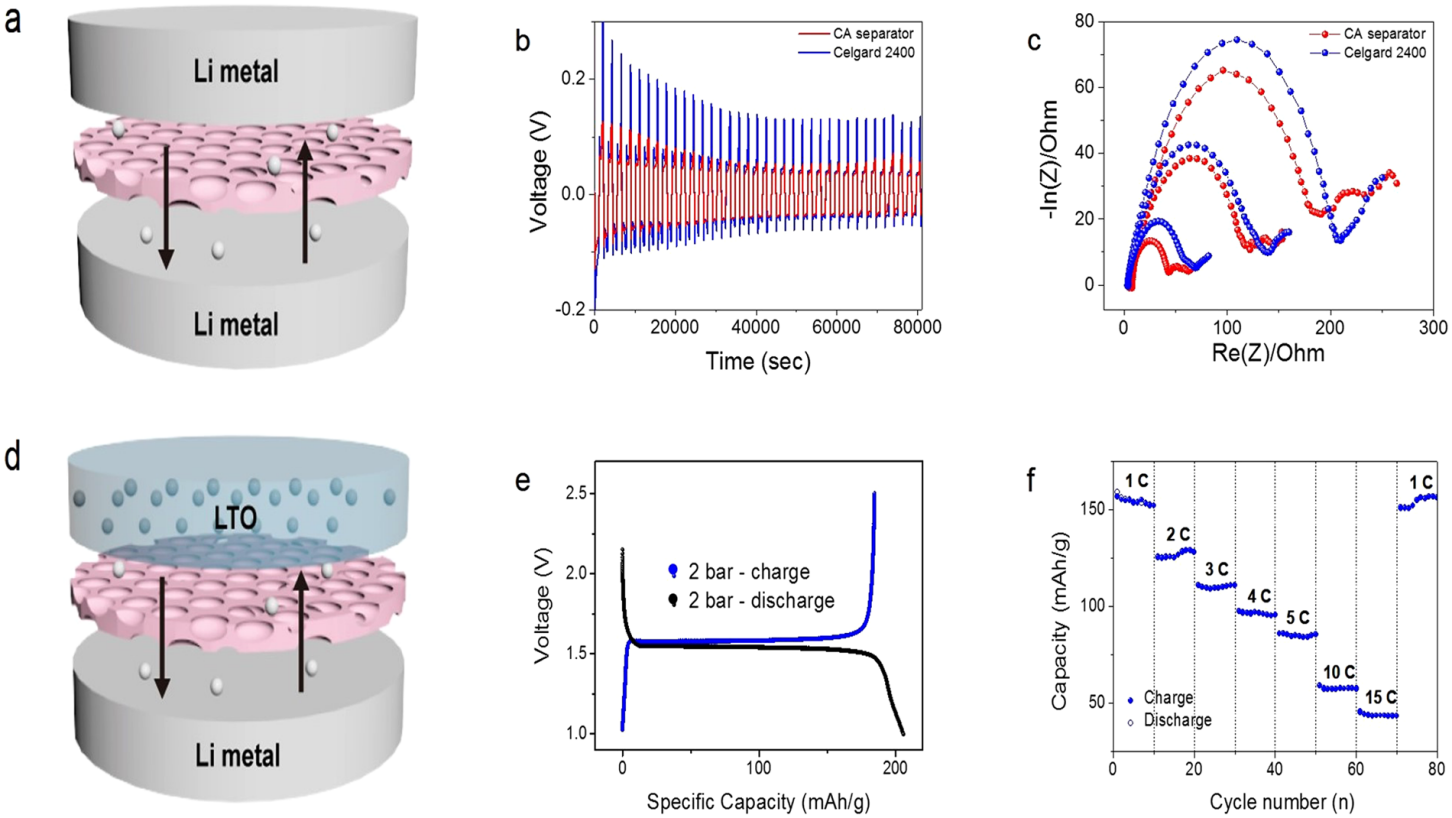
(**a**) Schematic representation of the Li metal/cellulose acetate (CA) separator/ Li metal symmetry cell architecture. (**b**) chronopotentiometry results of symmetry cell with the polymeric Celgard separator (blue line) and the 1:0.23 CA/Ni(NO_3_)_2_ polymer separator at a water pressure of 2 bar (red line). (**c**) The Nyquist plots of symmetry cells with Celgard (blue dots) and CA (red dots) separators as a function of cycle number (**d**) Schematic representation of the Li metal/CA separator/LTO half-cell architecture. (**e**) Galvanostatic discharge-charge profiles of half-cell with the 1:0.23 CA/Ni(NO_3_)_2_ polymer separator at a water pressure of 2 bar. (**f**) Rate performance of Li metal/CA separator/LTO half-cell.

The precisely controlled pore size of our CA polymer separators was characterized by the galvanostatic plating/stripping method from stacked Li metal/CA separator (after water pressure treatment)/Li Metal architecture, as shown in Fig. 9(a). For electrochemical measurements, a relatively low pressurized CA separator was applied because a high water pressure sample (ie 8 bar) often foamed unexpected large pores during the process. 1.3 M lithium hexafluorophosphate (LiPF_6_) in EC/DEC (50 v/50 v) with 10% FEC additive was used as an electrolyte for the cell test. In order to measure the electrochemical characteristics in the symmetry cells, we initially plated 0.5 C Li metal on the working electrode and stripped the same amount with a galvanostatic current of ±0.5 mA (Fig. 9(a)). Figure 9(b) shows the plating/stripping cycle potential profiles containing polymeric and CA separator after water pressure treatment at 2 bar. The polymeric Celgard separator shows a higher plating/stripping potential than the symmetry cell with the CA separator after water pressure treatment at 2 bar. In addition, the electrochemical impedance measurements of CA separator in Fig. 9(c) exhibit smaller passivation and charge transfer resistance than that of polymeric separator, indicating that the CA separator can effectively lower the resistance in the Li/Li symmetry cell system.

To extend our new CA separator for more realistic battery applications, we constructed a LTO/ CA separator (after water pressure treatment) /Li metal configuration battery with 1.3 M LiPF_6_ in EC/DEC (50 v/50 v) with a 10% FEC additive electrolyte, as shown in Fig. 9(d). The galvanostatic discharge/charge profile clearly exhibits stable discharging/charging plateaus at 1.54 V and 1.58 V, respectively (Fig. 9(e)). Using this half-cell configuration, we were also able to measure the various current rates from 1 C to 15 C, as shown in Fig. 9(f). Upon increasing the current rate of the cell, the average capacity monotonically decreased from 160 mAh/g to 50 mAh/g and completely recovered the average capacity at a 1 C rate. This stable battery operation suggests that the CA separator after water pressure treatment of 2 bar can sustain Li-ion exchange in the cell even at high current densities.

In conclusion, we utilized an inorganic complex in concert with isostatic water pressure treatment for a CA polymer. We found that when the CA/Ni(NO_3_)_2_·6H_2_O complex film was exposed to isostatic water pressure, continuous nanopores were generated in the polymer. The generated nanopores could be explained by the weakened polymer chains, which resulted from the plasticization effect of the incorporated Ni(NO_3_)_2_·6H_2_O into CA, resulting in the interconnected pores when water pressure was applied. The high electrolyte uptake ability of nanoporous CA polymer is carefully confirmed by experimental and MD simulation. Such a high Li affinity in CA separator allows stable Li-ion transfer in the half cell structure.

## Methods

### Separator fabrication

A 10% (w/w) solution of CA was obtained by dissolving CA in acetone/water (w/w 8:2). To prepare the polymer matrix, the amount of nickel(II) nitrate hexahydrate (Ni(NO_3_)_2_·6H_2_O) added to the polymer matrix was determined according to the mole ratio of the Ni ion to the monomeric unit of CA. At this stage, the final mixture was stirred for 2 h at room temperature. Subsequently, the solutions were cast about 200 µm thickness by using doctor blade to form a freestanding film on a glass plate and dried under atmospheric pressure for 12 h. The dried polymer matrix containing Ni(NO_3_)_2_·6H_2_O was then subjected to water pressures ranging from 2 to 8 bar. The water flux of the films with varying porosities was measured and expressed in units of L/m^2^h.

### Symmetry and half cell fabrication

The battery-grade 1.3 M LiPF_6_-EC/DEC (ethylene carbonate/diethyl carbonate) with a 10 wt% fluoroethylene carbonate (FEC) electrolyte was used for both the symmetry and half cell. The 300 μm thick pure Li metal and 5 μm thick Li metal on Cu foil were purchased from Wellcos Co. and Sidrabe Co., respectively. The active electrodes consisted of LTO, PVDF, and Super P (8:1:1) and were dissolved in NMP and subsequently coated on a Cu foil. The active electrodes were dried in a vacuum oven at 80 °C for 12 h. The PS (Celgard® 2400) separator was used as a reference separator. The 2032-type coin cells were used for symmetry and half-cell measurements. The coin cells were pressurized by using a crimping machine (Hohsen Corp). The battery cells were prepared in an Ar-filled glove box (<0.1 ppm O_2_ and H_2_O).

### MD simulation

For the molecular dynamics (MD) study, CA, EC and DEC were modeled (Figure S2 and Table S1) and two individual system of CA and a mixture of EC/DEC were separately constructed with 5 of 20 mer CA polymer and with 564 of EC and 310 of DEC molecules, respectively. Each system was relaxed with NPT (i.e. isothermal-isobaric ensemble) MD for 500 ps with the time step of 0.5 fs. Note that temperatures were set to be 500 K for annealing CA and 363 K for relaxing the EC/DEC mixture. Then, the two systems were systematically put together into the periodic box with the size of 40.6×40.6×137.1 Å^3^, which was subsequently relaxed by NPT MD for another 500 ps at 500 K and 1 atm. Finally, the layered system (Figs 6 and 7) was performed with NPT MD for 5 ns with the time step of 1 fs at 363 K and 1 atm. For the MD simulations, COMPASS II forcefield was used^35, 36^.

### Characterization

Fourier transform infrared (FT-IR) measurements were performed on a Varian FTS3100 spectrometer; 64–200 scans were averaged at a resolution of 4 cm^−1^. Thermogravimetric analysis (TGA) was performed with Mettler Toledo TGA devices at a heating rate of 10 °C/min. Scanning electron microscopy (SEM, JSM-5600LV, JEOL) was used to investigate the pores generated in the polymer. Additionally, a mercury porosimeter (Autopore IV9500 model, Micromeritics Inc.) was used to analyze the pore size and porosity generated in the polymer matrix. Electrochemical experiments were performed using a BioLogic VMP3 or a WonATech battery tester (WBCS3000L).

## Electronic supplementary material

supporting information
