# Supplementary material for: Facile control of nanoporosity in Cellulose Acetate using Nickel(II) nitrate additive and water pressure treatment for highly efficient battery gel separators
Source: Sci Rep. 2017 Apr 28;7:1287. doi: 10.1038/s41598-017-01399-8 (PMC5430623; doi:10.1038/s41598-017-01399-8)
Supplement: Supplementary file 1 — supporting information [file 41598_2017_1399_MOESM1_ESM.pdf]

Facile control of nanoporosity in Cellulose Acetate using Nickel(II) nitrate additive and water pressure treatment for highly efficient battery gel separators

Woong Gi Lee,[a,†] Do Hyeong Kim,[b,†], Woo Cheol Jeon,[b] Sang Kyu Kwak,[b,*], Seok Ju Kang,[b,*] and Sang Wook Kang[a,*]

[a] *Department of Chemistry, Sangmyung University, Seoul 110-743, Republic of Korea*

[b] *School of Energy and Chemical Engineering, Ulsan National Institute of Science and Technology (UNIST), Ulsan 689-798, Republic of Korea*

Supporting information

SEM image of CA porous membrane

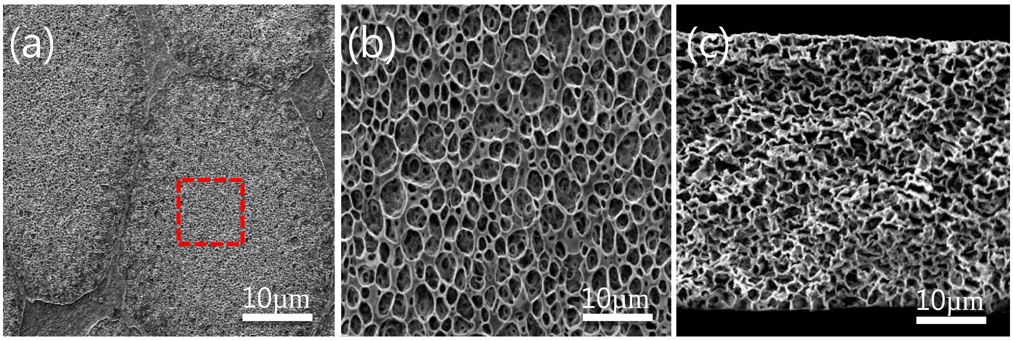

Figure S1. Planar scanning electron microscopy (SEM) images of CA surface morphology observed in 1:0.23 CA/ $Ni(NO_3)_2·6H_2O$ in acetone/water (w/w 8:2) at water pressures of 8 bar (a). The red dashed squares in (a) indicate the magnified areas shown in panes (b), respectively. Cross-sectional views of the samples subjected to water pressures of 8 bar (c).

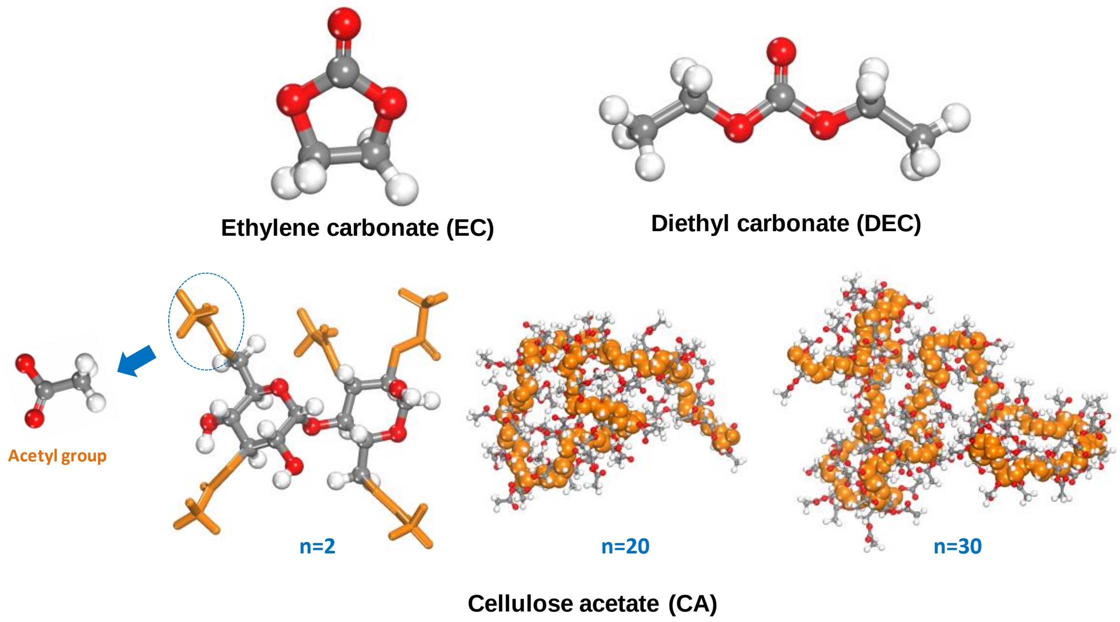

Figure S2. Molecules used for the simulation study. Orange sticks represent acetyl groups, which are included in CA. *n* designates the degree of polymerization (DP) of CA. Orange spheres represent the backbone atoms of CA at *n* = 20 and 30. Degree of substitution (DS) [1], which determines the number of acetyl groups in CA monomer, can be calculated with $DS = \frac{3.86 \times wt\%acetyl}{102.4 - wt\%acetyl}$. Since experimental *wt*% of acetyl group in CA is 39.8 *wt*%, DS is calculated to 2.45 so that we constructed the CA dimer to contain five acetyl groups. The degree of polymerization (DP) was set to 20 and 30, which showed a linear increase of the radius of gyration (R_g), to check the DP effect on the MD simulation. Atomic charges of CA were assigned using a grid-based method (CHELPG)[2] from density functional theory (DFT) calculation using B3LYP[3-4]/6-31+G**[5] functional and those of EC and DEC were obtained from OPLS-AA[6] and COMPASS II forcefield[7-8], respectively.

Table S1. Calculated density of the pure system of each molecule in the bulk phase. Note that CA is the dimer model. The values in parentheses are from the references[9-11].

(g/cc)	EC	Error(%)	DEC	Error(%)	CA	Error(%)
Density	1.304 (1.321)[9]	1.3	0.963(0.975)[10]	1.2	1.325(1.3)[11]	1.9

In order to validate the molecular models on our interest, we have constructed the pure system of each component (i.e. 100 molecules) in the bulk phase and performed NPT (i.e. isothermal-isobaric ensemble) MD simulations for 500 ps at 298 K for CA and DEC and at 313 K for EC. The pressure was set to 1 atm. The densities of the molecular models showed good agreements with experiments.

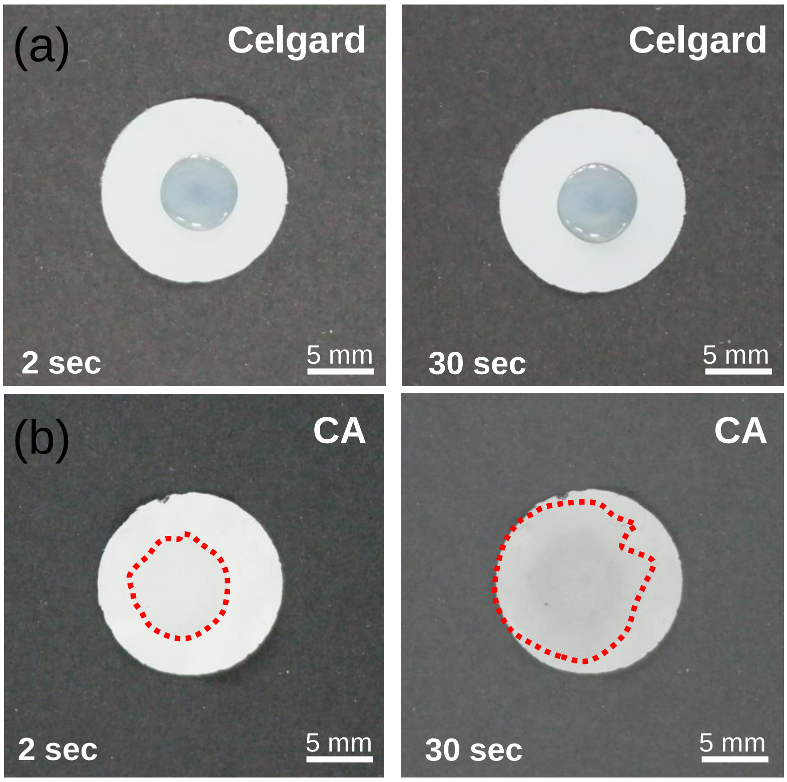

Figure S3. Photographs of (a) Celgard and (b) water-treated 1:0.23 CA/ $Ni(NO_3)_2{\cdot}6H_2O$ membranes after drop of 1.3 M $LiPF_6$- EC/DEC (50v/50v) with 10% FEC electrolyte.

Supplementary Note 1 - Estimation of Hildebrand solubility parameter and Flory-Huggins interaction parameter

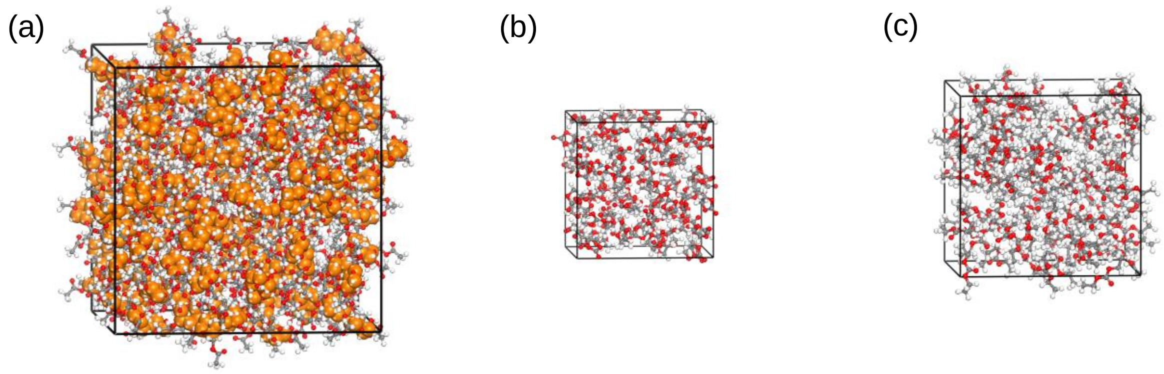

Figure S4. Model systems for calculating Hildebrand solubility parameters (δs) of (a) CA dimer, (b) EC, and (c) DEC. Each system consists of 100 molecules. Orange spheres represent backbone atoms in CA. Grey, red and while colors represent carbon, oxygen and hydrogen atoms, respectively.

In order to estimate Hildebrand solubility parameter[12] (δ), the bulk systems of CA, EC, and DEC were constructed as shown in Figure S5. Each NPT (i.e. isothermal-isobaric ensemble) MD simulation at 298 K, 323 K, and 363 K and 1 atm was run for 500 ps after energy minimization and relaxing of the system with the time step of 1.0 fs. The cohesive energy density (CED) (i.e. δ = (CED)$^{0.5}$) was obtained from the configurations of the system with the last 100 ps of the simulation by the equation as follows.

$$CED = \frac{E_{coh}}{V} \quad (1)$$

where V is the total volume of system and E_{coh} is the cohesive energy. The cohesive energy can be described as follows,

$$E_{coh} = -\langle E_{inter} \rangle = \langle E_{intra} \rangle - \langle E_{total} \rangle \quad (2)$$

where E_{inter} is intermolecular energy of all molecules, E_{intra} is intramolecular energy of all molecules and E_{total} is total energy of the system. The brackets indicate an ensemble average. Subsequently, Flory-Huggins interaction parameters[13-14] (χ_{ij}) based on Flory-Huggins theory were calculated as follows,

$$\chi_{ij} = \frac{V_S}{RT}(\delta_i - \delta_j)^2 \quad (1)$$

where V_S is the molar volume of the reference molecule, δ_i and δ_j are solubility parameters of different components i and j, R is the ideal gas constant, and T is temperature.

Supplementary Note 2 - Diffusivity

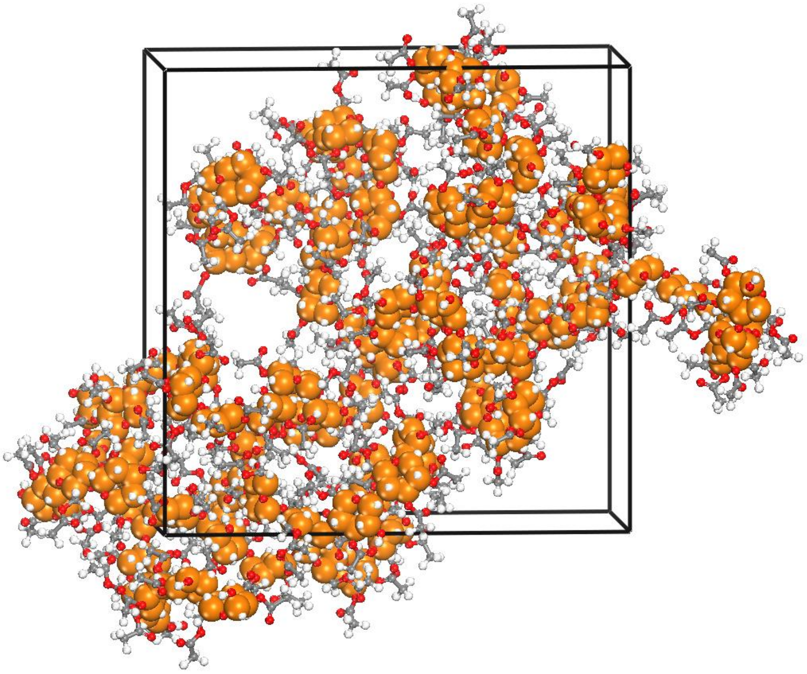

Figure S5. Model system containing 5 of 20mer CA in the bulk phase. Orange spheres represent backbone atoms in CA.

In order to check the degree of diffusion of CA when interacted with EC/DEC, the bulk system of CA (Figure S5) was constructed for the comparison while having the same number of CA as in the layered system (Figure 1). Note that the bulk CA system was subject to the same simulation condition of the layered system. The degree of diffusion was estimated by diffusivity (or diffusion coefficient), which can be calculated with the following expression,

$$D = \frac{1}{6N} \lim_{t \to \infty} \frac{d}{dt} \sum_{i=1}^{N} \left\langle \left[r_i(t) - r_i(0) \right]^2 \right\rangle \quad (2)$$

where N is the number of molecules, t is the simulation time, and r_{i} is the position vector of i th molecule.

References

1. Olabisi, O. Handbook of Thermoplastics; Marcel Dekker: New York, p 342 (1997).
2. Breneman, C. M.; Wiberg, K. B. *J. Comput. Chem.* **11** (3), 361-373 (1990).
3. Vosko, S. H.; Wilk, L.; Nusair, M. *Can. J. Phys.* 58, 1200 (1980).
4. Lee, C.; Yang, W.; Parr, R. G. *Phys. Rev. B.* **37**, 785 (1988).
5. Hariharan, P. C.; Pople, J. A. *Theor. Chim. Acta.* **28**, 213−222 (1973).
6. Cornell, W. D.; Cieplak, P.; Bayly, C. I.; Gould, I. R.; Merz, K. M.; Ferguson, D. M.; Spellmeyer, D. C.; Fox, T.; Caldwell, J. W.; Kollman, P. A. *J. Am. Chem. Soc.* **117**, 5179−5197 (1995).
7. Sun, H.; Jin, Z.; Yang, C. W.; Akkermans, R. L. C.; Robertson, S. H.; Spenley, N. A.; Miller, S.; Todd, S. M. *J. Mol. Model.* **22** (2) (2016).
8. Materials Studio 2016; BIOVIA Inc.: San Diego, CA, (2016).
9. Morita, M.; Asai, Y.; Yoshimoto, N.; Ishikawa, M. *J. Chem. Soc. Faraday. T.* **94** (23), 3451-3456 (1998).
10. Trenzado, J. L.; Romano, E.; Segade, L.; Caro, M. N.; Gonzalez, E.; Galvan, S. *J. Chem. Eng. Data.* **56** (6), 2841-2848 (2011).
11. Haas, D.; Heinrich, S.; Greil, P. *J. Mater. Sci.* **45** (5), 1299-1306 (2010).
12. Hildebrand, J. H.; Scott, R. L. Reinhold, New York, (1950).
13. Flory, P. J. *J. Chem. Phys.* **9**, 660 (1941).
14. Huggins, M. L. *J. Chem. Phys.* **9**, 440 (1941).
